# Placebo Effects Are Small on Average in the 7.5% CO_2_ Inhalational Model of Generalized Anxiety

**DOI:** 10.1093/ijnp/pyae019

**Published:** 2024-04-05

**Authors:** Nathan T M Huneke, Cosmina Cross, Harry A Fagan, Laura Molteni, Naomi Phillips, Matthew Garner, David S Baldwin

**Affiliations:** Southern Health National Health Service Foundation Trust, Southampton, UK; Clinical and Experimental Sciences, Faculty of Medicine, University of Southampton, UK; University Department of Psychiatry, Academic Centre, College Keep, Southampton, UK; Southern Health National Health Service Foundation Trust, Southampton, UK; Southern Health National Health Service Foundation Trust, Southampton, UK; Clinical and Experimental Sciences, Faculty of Medicine, University of Southampton, UK; University Department of Psychiatry, Academic Centre, College Keep, Southampton, UK; Clinical and Experimental Sciences, Faculty of Medicine, University of Southampton, UK; University Department of Psychiatry, Academic Centre, College Keep, Southampton, UK; Solent National Health Service Trust, Southampton, UK; Center for Innovation in Mental Health, School of Psychology, Faculty of Environmental and Life Sciences, University of Southampton, UK; University Department of Psychiatry, Academic Centre, College Keep, Southampton, UK; University Department of Psychiatry and Mental Health, University of Cape Town, Cape Town, South Africa; Southern Health National Health Service Foundation Trust, Southampton, UK; Clinical and Experimental Sciences, Faculty of Medicine, University of Southampton, UK; University Department of Psychiatry, Academic Centre, College Keep, Southampton, UK

**Keywords:** Anxiety disorders, anxiety, placebo effect, placebo response

## Abstract

**Background:**

Anxiety disorders are highly prevalent and socio-economically costly. Novel pharmacological treatments for these disorders are needed because many patients do not respond to current agents or experience unwanted side effects. However, a barrier to treatment development is the variable and large placebo response rate seen in trials of novel anxiolytics. Despite this, the mechanisms that drive placebo responses in anxiety disorders have been little investigated, possibly due to low availability of convenient experimental paradigms. We aimed to develop and test a novel protocol for inducing placebo anxiolysis in the 7.5% CO_2_ inhalational model of generalized anxiety in healthy volunteers.

**Methods:**

Following a baseline 20-minute CO_2_ challenge, 32 healthy volunteers were administered a placebo intranasal spray labelled as either the anxiolytic “lorazepam” or “saline.” Following this, participants surreptitiously underwent a 20-minute inhalation of normal air. Post-conditioning, a second dose of the placebo was administered, after which participants completed another CO_2_ challenge.

**Results:**

Participants administered sham “lorazepam” reported significant positive expectations of reduced anxiety (*P *= .001), but there was no group-level placebo effect on anxiety following CO_2_ challenge post-conditioning (*P*s > .350). Surprisingly, we found many participants exhibited unexpected worsening of anxiety, despite positive expectations.

**Conclusions:**

Contrary to our hypothesis, our novel paradigm did not induce a placebo response, on average. It is possible that effects of 7.5% CO_2_ inhalation on prefrontal cortex function or behavior in line with a Bayesian predictive coding framework attenuated the effect of expectations on subsequent placebo response. Future studies are needed to explore these possibilities.

**Significance Statement**

Anxiety disorders are highly prevalent and socio-economically costly. We need novel pharmacological treatments for these disorders because many patients do not respond to current agents or experience unwanted side effects. However, a barrier to treatment development is the variable and large placebo response rate seen in trials of novel anxiolytics. Despite this, the mechanisms that drive placebo responses in anxiety disorders have been little investigated. In this study, we tested a novel conditioning procedure, utilizing the 7.5% CO_2_ inhalational model of generalized anxiety, designed to induce placebo anxiolytic responses in healthy volunteers. Unexpectedly, we did not find a group-level placebo effect on anxiety. Our study highlights important questions about the psychological basis for placebo effects and further validates the use of the 7.5% CO_2_ model for evaluating new anxiolytic compounds in proof-of-principle studies.

## INTRODUCTION

Anxiety disorders are the most common mental disorders, with an estimated current prevalence of 7% to 14% and lifetime prevalence of 10% to 16% (Wittchen et al., 2011; Baxter et al., 2013; Remes et al., 2016). Globally, anxiety disorders are the sixth greatest cause of nonfatal health loss (World Health Organization, 2017). Anxiety disorders cause impairments in social and occupational functioning and are frequently comorbid with other psychiatric and physical illnesses (Hendriks et al., 2016; Bokma et al., 2017). As a result, anxiety disorders are associated with marked socioeconomic and healthcare burden (Olesen et al., 2012). Currently, initial psychological treatments for anxiety disorders are limited in availability, medications cause unwanted side effects, and remission rates following first-line treatments are only 40% to 55% (Bereza et al., 2012; Springer et al., 2018). Considering their socioeconomic burden, there is a need to develop improved treatments for anxiety disorders.

Despite this clear need, novel drug discovery in the field of anxiety disorders has been generally unfruitful. Nearly 1500 novel drug treatments have been tested in the past 50 years, many of which showed initial promise, but few have translated into effective treatments in humans (Griebel and Holmes, 2013). Contributors to this poor return are likely to include poor understanding of the underlying neurobiology and poor validity of preclinical models of psychiatric disease leading to a “mismatch” between preclinical and clinical studies for novel treatments (Griebel and Holmes, 2013; Stewart et al., 2015; Monteggia et al., 2018). An additional factor to consider is the large placebo response rate seen in anxiolytic drug trials. “Placebo response” is the health improvement that occurs after administration of an inactive treatment (Evers et al., 2018).

Meta-analyses in anxiolytic trials demonstrate a within-group placebo effect size of 1.03 to 1.29, which is approximately 75% of the effect size for medication, and this effect size has increased over time (Bandelow et al., 2015; De Vries et al., 2016). Further, placebo effects are more variable than active medication effects in antidepressant trials (Iovieno and Papakostas, 2012). The sources of this variation are not fully understood but are likely partially due to statistical artifact or nonspecific effects, including regression to the mean, sampling biases, or nonspecific benefits from interactions with healthcare staff (Ernst and Resch, 1995; Miller and Rosenstein, 2006). Additionally, there is evidence that the administration of placebos can lead to genuine physiological changes in biological systems, including the immune, dopaminergic, and endogenous opioid systems (De La Fuente-Fernandez, 2001; Benedetti et al., 2011; Albring et al., 2012; Wager and Atlas, 2015). Since placebo effect size is changeable and factors that influence it are not fully understood, it is currently challenging to predict how large it will be in any given clinical trial (Huneke, 2022). This can affect the ability of a clinical trial to distinguish efficacy of active treatment from placebo, hindering psychotropic drug development over time (Enck et al., 2013; Huneke et al., 2020a; Huneke, 2022; Correll et al., 2023). Therefore, improved understanding of the mechanisms that contribute to placebo responses in anxiety disorders is needed.

Placebo analgesia, the reduction of pain through placebo mechanisms, has been investigated in detail (for reviews, see Wager and Atlas, 2015; Ashar et al., 2017). There are numerous well-established experimental paradigms that consistently induce placebo analgesic effects in healthy volunteers and in patients (Meissner et al., 2011). By contrast, manipulation of anxiety through placebo mechanisms has been little investigated. Only 1 paradigm has been developed explicitly with the aim of experimentally inducing placebo *anxiolysis* (as opposed to reducing *distress*) in healthy volunteers (Meyer et al., 2015, 2019). In this paradigm, healthy volunteers undergo an unpredictable threat of shock task. In some runs, a placebo with verbal suggestions that it is an anxiolytic drug is administered, while in other runs they are given a placebo and told it is inert. In all 3 experiments published with this paradigm, placebo globally reduced subjective fear and skin conductance responses, regardless of whether the trial was threat or nonthreat (Meyer et al., 2015, 2019). As a result, it is unclear whether this represents placebo anxiolysis. Furthermore, the paradigm is confounded by the fact that the threatening stimulus is painful, potentially recruiting placebo analgesic mechanisms. Finally, this paradigm involves phasic threat, to which an anxiety response would be considered functional, as opposed to the dysfunctional anxiety seen in anxiety disorders (Robinson et al., 2013). An anxiogenic stimulus that causes dysfunctional anxiety would be more clinically relevant.

It is possible to induce features similar to those seen clinically in anxiety disorders through the use of laboratory-based experimental medicine models in healthy volunteers (Baldwin et al., 2017). One such model is the 7.5% CO_2_ inhalational model of generalized anxiety. Inhalation of air “enriched” with 7.5% CO_2_ (or 7.5% “CO_2_ challenge”) mimics the subjective, autonomic, and neurocognitive features of generalized anxiety disorder (Bailey et al., 2005; Garner et al., 2011). In addition, anxiety in this model can be ameliorated by standard pharmacological and psychological treatments for generalized anxiety disorder (Bailey et al., 2007; Ainsworth et al., 2015). Because this model is treatment responsive, has construct validity for clinical anxiety, and allows for control of the anxiogenic stimulus intensity, we hypothesized it could be an ideal basis for development of a novel experimental placebo anxiolytic paradigm.

In the current study, we tested whether it was possible to induce a clinically relevant placebo response in the 7.5% CO_2_ inhalational model of generalized anxiety. If this was possible, then such a paradigm would allow further experiments to delineate the mechanisms that drive placebo anxiolysis, ultimately to improve clinical trial design, enhance effectiveness of current treatments, and identify novel disorder mechanisms and therapeutic targets (see Huneke et al., 2020a; Huneke, 2022). Placebo effects are maximal when verbal suggestions and learning are combined (Bartels et al., 2014; Ashar et al., 2017). Therefore, we designed a paradigm that pairs a “sham” treatment with verbal suggestions and learning. Our design is similar to paradigms previously used to interrogate mechanisms of placebo analgesia (Watson et al., 2009; Meissner et al., 2011; Huneke et al., 2013). To summarize, we aimed to induce a placebo response to sham “lorazepam” in healthy volunteers through a conditioning procedure whereby participants were given “lorazepam” followed by sham CO_2_ (normal air), expecting participants would learn the treatment was anxiolytic. Following this, participants underwent a 7.5% CO_2_ inhalation to test whether placebo anxiolysis was induced. We expected participants to experience less CO_2_-related anxiety after “lorazepam” administration. We compared outcomes with a fully informed control group, whose purpose was to account for nonspecific effects over time.

## METHODS

This study was reviewed and approved by the Ethics and Research Governance Office at the University of Southampton (reference: 52726). Information regarding our aim to study the placebo effect was initially withheld; however, on completing the study participants were debriefed, and fully informed consent was sought a second time. Participants were informed that they could decline and their data would be destroyed. No participants withdrew consent.

### Participants

**Figure 1.**
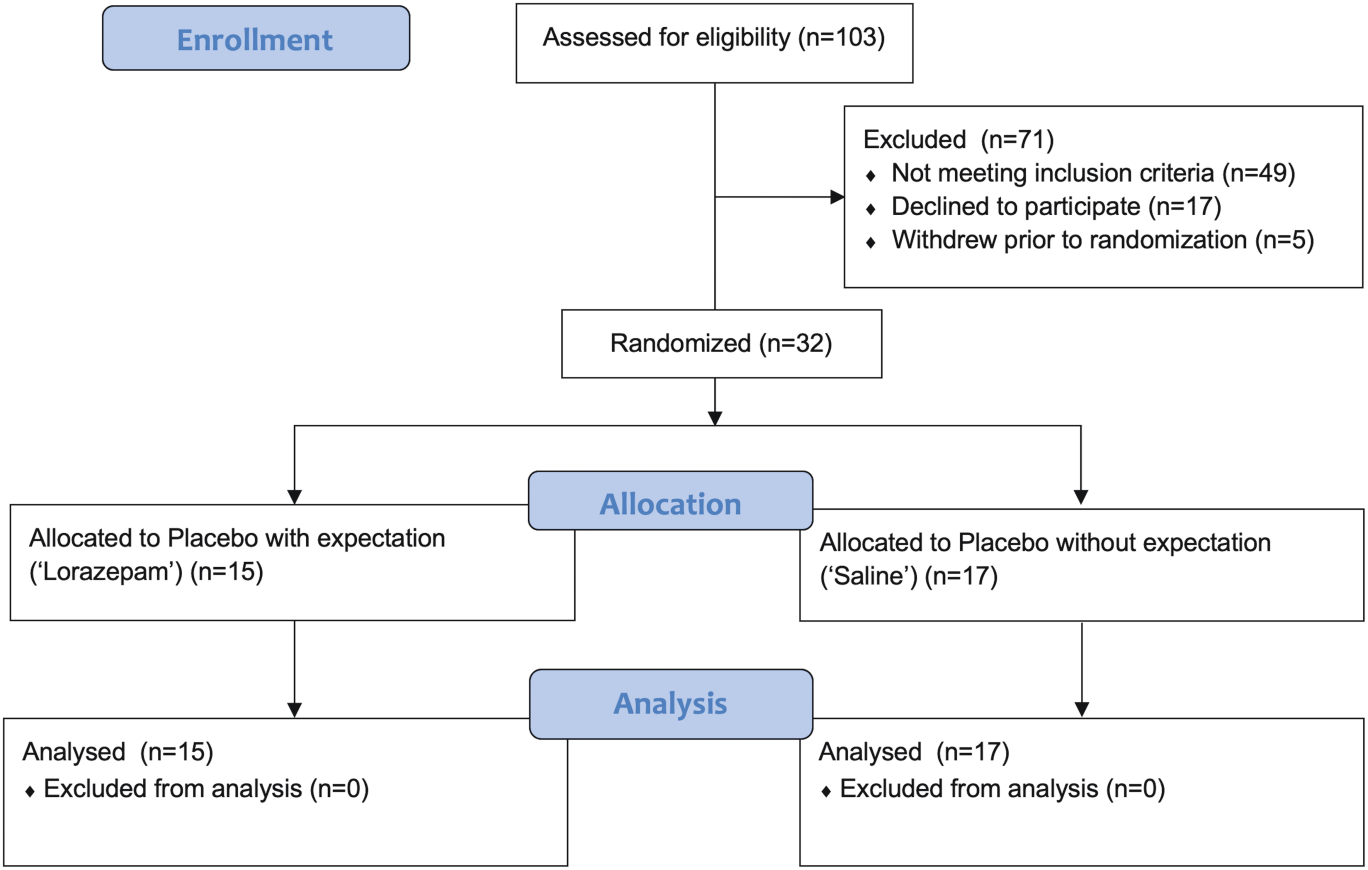
CONSORT diagram.

Thirty-two healthy volunteers (aged 18–55 years) were recruited from the community via advertisements (see Figure 1). They were offered £15 or course credits (psychology students) as reimbursement for participating. Exclusion criteria were current or lifetime history of psychiatric illness as assessed by the Mini International Neuropsychiatric Interview for DSM 5 (Sheehan et al., 1998); body mass index <18 or >28 kg/m^2^; chronic physical illness; regular smokers (>6 cigarettes per day); medication use in the previous 8 weeks; current alcohol intake >21 units per week; or illicit drug misuse (more than twice in the past 12 months).

### Sham Treatment

Potential participants were informed that the purpose of the study was to assess the effects of “intranasal lorazepam” administered as a nasal spray. Lorazepam is a licensed treatment for anxiety disorders in the United Kingdom but is not available as a nasal spray. The spray administered to participants was instead a normal saline nasal spray with no active ingredients. We chose a placebo nasal spray over other modalities (e.g., a pill) because more invasive placebos cause larger placebo effects than non-invasive alternatives such as a pill (Liu, 2017). Furthermore, so-called active placebos that generate side effects are more effective than inactive placebos (Rief and Glombiewski, 2012), possibly due to the expectations these side effects engender (Ashar et al., 2017). A nasal spray is somewhat invasive and causes “side effects” in terms of mild irritation or itching in the nostril. A placebo nasal spray has successfully engendered placebo effects in previous studies (Meyer et al., 2015; Glombiewski et al., 2019; Guevarra et al., 2020; Rebstock et al., 2020; Göhler et al., 2021).

### Experimental Placebo Procedure

**Figure 2.**
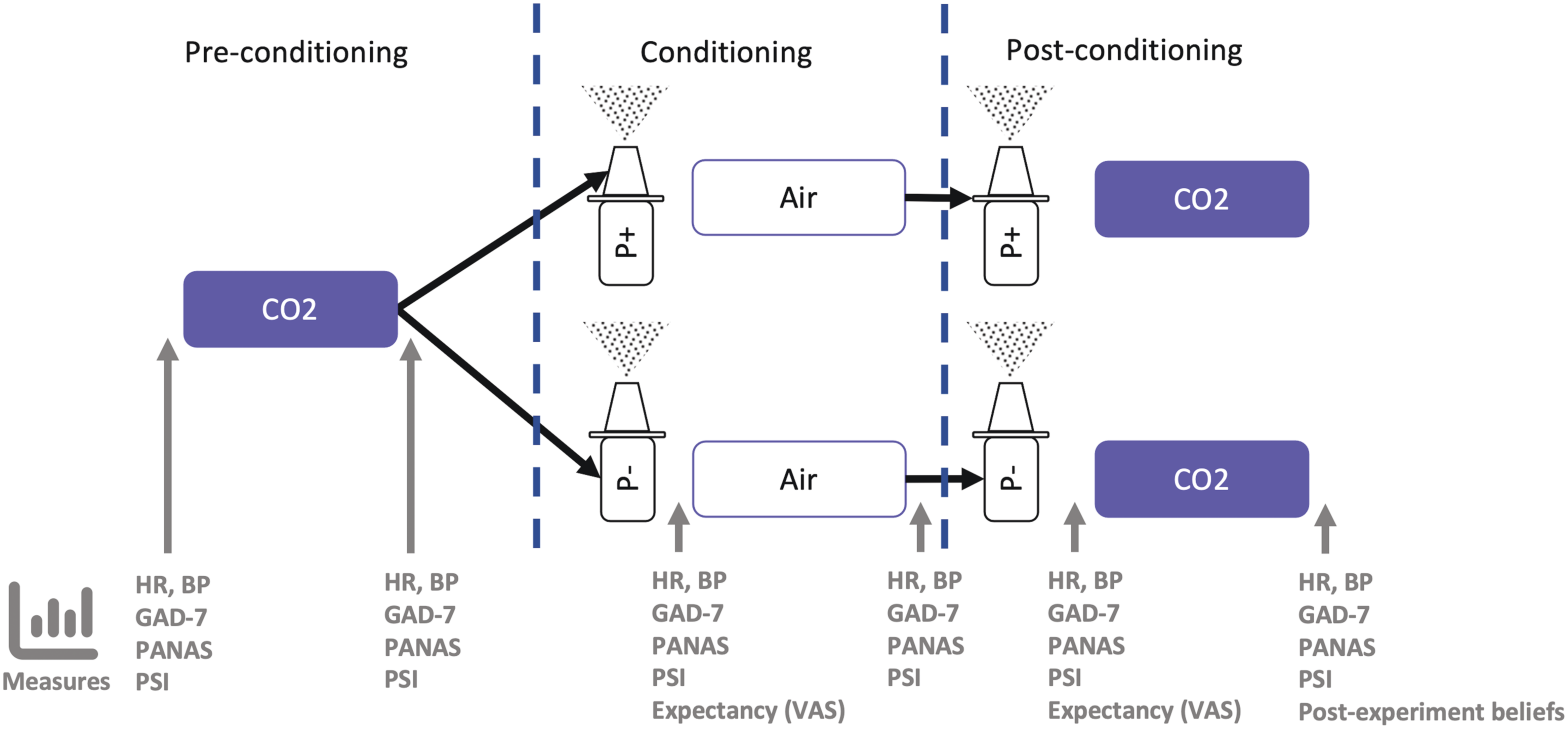
Schematic of experimental placebo anxiolysis paradigm. After baseline CO_2_ challenge (pre-conditioning), participants were randomized to either the placebo with expectation (P+, “lorazepam”) or placebo without expectation group (P−, “saline”). They then underwent a conditioning procedure, in which the participants inhaled air in place of 7.5% CO_2_ gas mixture. In the post-conditioning period participants again underwent CO_2_ challenge. Outcome measures were taken before and after each inhalation period. Abbreviations: BP, blood pressure; GAD-7, generalized anxiety disorder-7 questionnaire; HR, heart rate; PANAS, Positive and Negative Affect Schedule; PSI, panic symptom inventory; VAS, visual analogue scale.

Before participation, potential volunteers were given information about lorazepam, including that it is used as an anxiolytic and its possible side effects. Eligible participants attended an experimental session, comprising 3 segments: baseline pre-conditioning, conditioning and post-conditioning (see Figure 2). In the baseline pre-conditioning segment, participants underwent a 7.5% “CO_2_ challenge.” Air augmented with 7.5% CO_2_ (21% O_2_, balance N_2_) was administered through an oronasal face mask for 20 minutes, as in previous studies (Garner et al., 2011; Ainsworth et al., 2015; Huneke et al., 2020b).

Following the baseline CO_2_ challenge, participants were randomized to 1 of 2 groups: either placebo with expectation or placebo without expectation. In the placebo with expectation group, participants took 2 doses of the “sham intranasal lorazepam” spray. The spray was administered with verbal information that it would work within seconds, as the nose is directly connected with the brain, and the spray would have a duration of action of 20–30 minutes. After receiving the spray, participants were told they would now repeat the CO_2_ challenge for 20 minutes. Instead, the experimenter surreptitiously changed the inhaled gas to normal air. The purpose of this deception was to condition the participants to believe the spray possessed anxiolytic properties. In the post-conditioning segment, participants again took 2 doses of the “lorazepam” spray before undergoing another 20-minute CO_2_ challenge. They were told that this was to investigate the effects of repeated doses of “intranasal lorazepam.” For simplicity, this group is referred to as the “lorazepam” group.

The placebo without expectation group underwent the same procedure, except they received truthful instructions throughout. After the pre-conditioning segment, they took 2 doses of a nasal spray labelled “saline.” This was accompanied by verbal information that the spray contained normal saline only and should not have any effect on subjective anxiety. These participants were also informed that the change to normal air in the second inhalation period would be the cause of any reductions in anxiety. In the post-conditioning segment, these participants again took 2 doses of the saline nasal spray and were then told that the next inhalation would again involve air enriched with 7.5% CO_2_, likely causing them to feel anxious. The purpose of this group was to control for the effects of repeated exposure to CO_2_ challenge as well as any other nonspecific “apparent” placebo effects (such as natural relaxation) that occurred during the experiment. For simplicity, this group is referred to as the “saline” group.

Most study procedures were carried out by an investigator who was blinded to group assignment. However, the “sham” treatments and instructions were given by an unblinded independent investigator while the blinded investigator was absent from the room. We blinded the investigator to reduce the risk of response bias, which is a potential confounder in placebo studies (Allan and Siegel, 2002; Hróbjartsson et al., 2011).

### Measures

#### Baseline Measures

Both placebo efficacy and response to CO_2_ challenge have been associated with trait personality and demographic variables. These include but are not limited to trait anxiety and affect (Eke and McNally, 1996; Geers et al., 2020; Kern et al., 2020), dispositional optimism (Morton et al., 2009; Kern et al., 2020), and anxiety sensitivity (Eke and McNally, 1996; Olatunji et al., 2009).

To ensure that the groups were balanced for potential predictors of placebo and CO_2_ responsiveness, we took several demographic and personality measures at baseline: age, biological sex, body mass index, heart rate, blood pressure, the Hospital Anxiety and Depression Scale (Zigmond and Snaith, 1983), the Penn State Worry Questionnaire (Meyer et al., 1990), the Intolerance of Uncertainty Scale (Buhr and Dugas, 2002), the Anxiety Sensitivity Index (Peterson and Reiss, 1992), the Revised Life Orientation Test (Scheier et al., 1994), the Locus of Control questionnaire (Rotter, 1966), and a modified version of the Generalized Anxiety Disorder 7-item (GAD-7) questionnaire (Spitzer et al., 2006), where each question was accompanied by a visual analogue scale ranging from “not at all” to “nearly every day.”

#### CO_2_ Outcome Measures

Outcome measures were taken before and after each inhalation. Subjective state anxiety was measured with a modified version of the GAD-7 (Spitzer et al., 2006), where, as with the baseline GAD-7, each question was represented by a visual analogue scale; but the scale values now ranged from “not at all” to “all of the time,” and the participant was asked to consider their anxiety over the previous 20 minutes. This questionnaire is brief, highly applicable to diagnostic criteria for generalized anxiety disorder, and the modification to include visual analogue scales means it is sensitive to change over time (Garner et al., 2011; Huneke et al., 2020b). Psychological and somatic symptoms of anxiety were further measured through the panic symptom inventory (Clark and Hemsley, 1982; Nutt et al., 1990). This is a 34-item scale that examines the psychological (e.g., feeling anxious, out of control, that they were dying) and somatic (e.g., heart pounding, breathlessness, muscle tension) features of panic attacks. Subjective changes in mood were assessed through the Positive and Negative Affect Schedule (Watson et al., 1988). We additionally measured autonomic stress through heart rate and blood pressure measurements taken with an automated sphygmomanometer (Omron-M6, Medisave, UK).

#### Expectations

We measured conscious expectations of therapeutic benefit from the spray immediately before the conditioning period (air inhalation) and immediately before the post-conditioning CO_2_ challenge. Participants were instructed to rate their answer to the question “how much do you expect this spray to reduce your anxiety by during the next inhalation?” on a 10-cm visual analogue scale ranging from “not at all” to “very much.” To check whether expectations remained consistent following conditioning, we also assessed post-experiment beliefs about the “sham” treatment using an adapted version of the therapy credibility questionnaire (Borkovec and Nau, 1972). Three items from this questionnaire were adapted to measure beliefs about the efficacy of the “sham” treatment and read as follows:

1. How confident are you that this treatment can successfully eliminate anxiety?
2. How confident would you be in recommending this treatment to a friend who suffers with anxiety in certain situations?
3. If you suffered with anxiety, would you be willing to have this treatment?

Each item was scored using a Likert scale from 1 to 5, with higher scores indicating greater beliefs about efficacy. This questionnaire was completed in the presence of the unblinded independent researcher to allow participants to be honest in their answers without accidental unblinding of the blinded investigator.

### Statistical Analysis and Power Calculation

Our outcomes of interest were changes in anxiety, mood, and autonomic measures over the course of the procedure. We hypothesized that the “lorazepam” group would exhibit a placebo effect: a significant reduction in CO_2_ outcome measures from pre- to post-conditioning and significantly decreased outcomes compared with the “saline” group post-conditioning.

The effect size of placebo is known to be smaller in clinical trials compared with the effect sizes seen in experimental placebo studies (Vase et al., 2002). Meta-analyses have shown that the average effect size of placebo analgesia in healthy volunteers is g = 1.24 (Forsberg et al., 2017), and in studies combining verbal suggestion with behavioral conditioning it is d = 1.48 (Vase et al., 2002). For this study, we recruited 32 participants, which provided 80% power to detect an effect size d* > *1.03 with an alpha level of 0.05 (2-tailed). The study was therefore powered to detect a placebo effect size comparable with that seen in the literature for similar experimental placebo studies.

Statistical analysis was carried out using Jamovi version 1.6.23.0 (https://www.jamovi.org) (The Jamovi Project, 2021). Baseline characteristics were compared between groups with independent samples *t* tests for continuous data or chi-squared tests for dichotomous data. Statistical analysis of CO_2_ outcome measures and change in expectations was carried out through mixed-model ANOVA with repeated measures. In all models, time was the within-subject factor and group was the between-subject factor. Significant results were explored further through post hoc *t* tests. The post-experiment therapy credibility questionnaire was analyzed through an independent samples *t* test.

## Results

### Baseline Characteristics

Baseline characteristics for each group are summarized in Table 1. These baseline characteristics and personality traits were broadly similar between groups.

**Table 1.** Baseline Characteristics

Measure	“Lorazepam”	“Saline”
n	15	17
Age	21.47 ± 1.96	19.71 ± 3.08
Females	10 (67%)	11 (65%)
BMI	23.03 ± 2.37	22.17 ± 2.50
Modified GAD-7	12.14 ± 8.75	15.54 ± 10.95
HADS-A	3.13 ± 1.92	3.29 ± 1.93
HADS-D	0.53 ± 0.74	1.82 ± 1.91
ASI	12.07 ± 7.16	12.82 ± 7.79
IUS	50.87 ± 12.59	48.12 ± 12.05
LOTR	17.33 ± 3.06	16.65 ± 3.86
LOC	12.33 ± 3.96	11.29 ± 3.92
PSWQ	39.20 ± 9.28	38.76 ± 10.10
Systolic BP (mmHg)	120.47 ± 11.86	113.18 ± 9.89
Diastolic BP (mmHg)	74.73 ± 9.54	71.35 ± 10.50
Pulse rate (bpm)	70.33 ± 14.17	75.53 ± 14.04

Abbreviations: ASI, Anxiety Sensitivity Index; BMI, body mass index; BP, blood pressure; bpm, beats per minute; GAD-7, Generalized Anxiety Disorder-7 questionnaire; HADS, Hospital Anxiety and Depression Scale; IUS, Intolerance of Uncertainty Scale; LOC, Locus of Control questionnaire; LOTR, Life Orientation Test Revised; mmHg, millimeters of mercury; PSWQ, Penn State Worry Questionnaire. Values are mean ± SD for continuous variables and count (%) for dichotomous variables.

### Effects of CO_2_ Challenge

**Figure 3.**
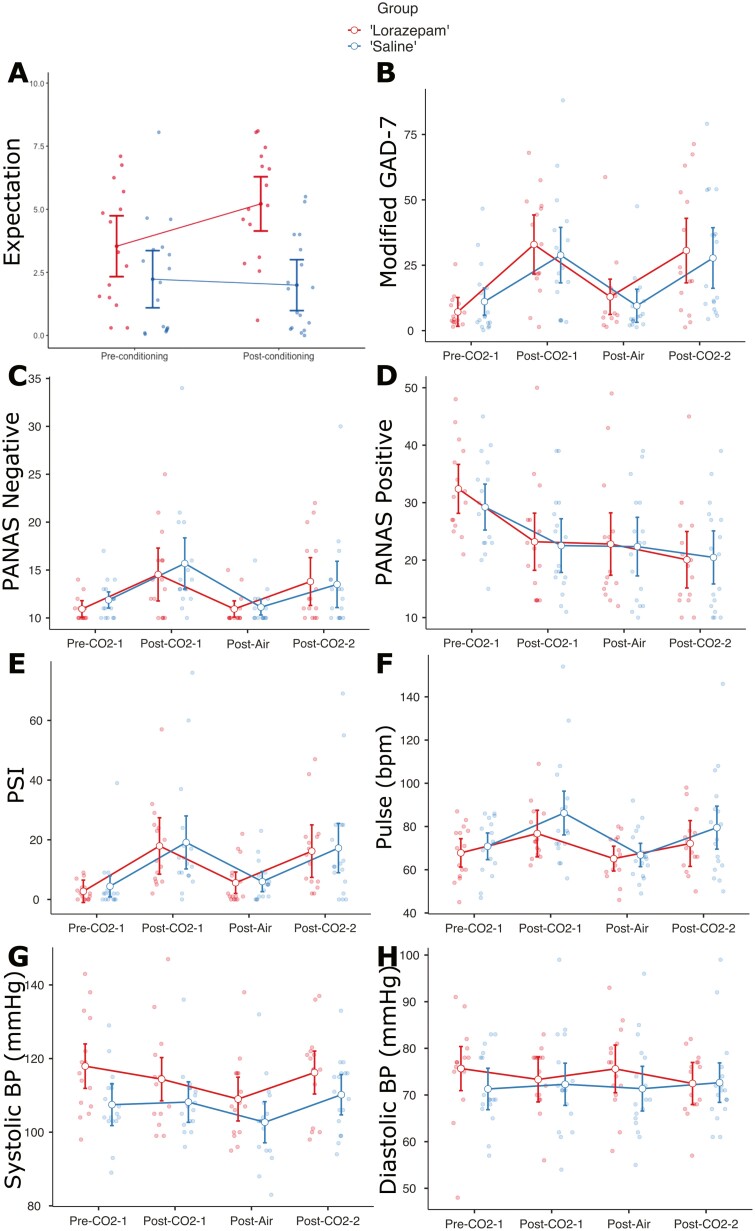
Graphs showing change in expectations, and in anxiety, mood and autonomic CO_2_ outcome measures over the course of the experiment. Points represent estimated marginal means and error bars represent 95% confidence intervals. The “lorazepam” group is shown in red and the “saline” group is shown in blue. Abbreviations: BP, blood pressure; bpm, beats per minute; GAD-7, Generalized Anxiety Disorder-7 questionnaire; mmHg, millimeters of mercury; PANAS, Positive and Negative Affect Schedule; PSI, Panic Symptoms Inventory; VAS, visual analogue scale.

Subjective and autonomic outcome measures of the CO_2_ challenge were analyzed through mixed-model ANOVA. There was a significant effect of time in all outcome measures except diastolic blood pressure. Following each 7.5% CO_2_ inhalation, subjective anxiety, pulse rate, and negative affect were significantly increased and positive affect significantly decreased, consistent with induction of anxiety (see Figure 3; supplementary Table 1).

### Expectations

We measured expectations prospectively (from pre- to post-conditioning) using a visual analogue scale and analyzed these data through mixed-model ANOVA with repeated measures. This revealed a significant effect of group (F_(1,30)_  = 12.72, *P* = .001, η_p_^2^ = 0.30) and a significant time*group interaction (F_(1,30)_ = 4.91, *P* = .035, η_p_^2^ = 0.14). Post-hoc tests showed these effects were driven by a significant increase in expectation from pre- to post-conditioning in the “lorazepam” group only (mean difference = 1.68, t_(30)_ = 2.66, *P* = .012) and a significant difference in expectation between groups post-conditioning (mean difference = 3.22, t_(30)_ = 4.46, *P* < .001; see Figure 3B). Beliefs about treatment credibility were also assessed at the end of the study. There was a significant difference in this measure between groups, with the “lorazepam” group rating the treatment as significantly more credible (mean difference = 2.28, t_(30)_ = 2.22, *P* = .034, d = 0.79). Overall, these results suggest that the conditioning paradigm induced a significant expectation of therapeutic benefit, which appeared to persist following the post-conditioning CO_2_ challenge.

### Effects of “Sham” Lorazepam

We analyzed the effects of “sham” lorazepam through mixed-model ANOVA. There were no significant time*group interactions in any outcome measure (Fs < 1.08, *P*s > .350), suggesting that mean outcomes were similar in both groups across the experiment (see Figure 3; supplementary Table 1). There was an effect of group on systolic blood pressure, but this appeared to be driven by a significant difference in systolic blood pressure between groups before the first inhalation (mean difference = 10.46, 95% CI = [2.17, 18.75], t_(30)_ = 2.58, *P* = .015). Following the first inhalation, there were no significant differences in systolic blood pressure between the groups (t_(30)_s < 1.60, *P*s > .110).

The effect sizes of 7.5% CO_2_ inhalation on subjective and autonomic anxiety were large in this study (η_p_^2^ ranged from 0.16 to 0.56). However, the effect size of the time*group interaction (i.e., of placebo conditioning) was small in all outcome measures (η_p_^2^ < 0.03). To understand the potential relevance of this effect, we conducted post-hoc between-group comparisons in outcomes following the post-conditioning CO_2_ inhalation and estimated the sample size required per group to achieve 80% power to detect placebo anxiolysis resulting from “sham” lorazepam administration. Minimum sample sizes ranged between 116 to 39 246 per group (see supplementary Table 2), suggesting that placebo effects would likely be undetectable with sample sizes that would usually yield significant treatment effects in proof-of-concept studies using the CO_2_ model.

### Exploratory Analyses of Effect of Expectations on Placebo Anxiolysis

At the group level, there appeared to be no evidence for placebo effects. However, it was possible that subgroups of participants exhibited differing responses, and that these might have been related to expectations. To investigate this, we calculated the “CO_2_ reactivity” for each participant in each CO_2_ inhalation by subtracting pre-inhalation values from post-inhalation values. Larger scores therefore indicate a greater reactivity to CO_2_ challenge. We then calculated how CO_2_ reactivity changed from pre-conditioning to post-conditioning by subtracting pre-conditioning reactivity from post-conditioning reactivity. Thus, negative values represent a reduction in reactivity (potentially placebo anxiolysis), while positive values represent an increase in reactivity over time.

There were no significant differences between the groups in change in CO_2_ reactivity for any outcome measure (all t_(30)_s < 1.60, *P*s > .130). Next, we correlated change in CO_2_ reactivity with expectations post-conditioning in the “lorazepam” group and “saline” group separately. In the “lorazepam” group and in subjective measures only, these correlations showed weak to moderate trend relationships between higher expectation post-conditioning and reduced CO_2_ reactivity (r’s −0.37 to −0.47, *P*s < .180). In contrast, there was either no relationship or a relationship in the opposite direction in autonomic outcome measures and in all measures in the “saline” group (see supplementary Figure 1). Interestingly, we also noted that several participants in the “lorazepam” group experienced increases in CO_2_ reactivity post-conditioning (see supplementary Figure 2).

## Discussion

The aim of this study was to test whether a novel experimental placebo paradigm might consistently induce placebo anxiolysis in healthy participants. Contrary to our hypotheses, the subjective and autonomic effects of CO_2_ challenge were very similar in the first and second CO_2_ inhalations (i.e., did not reduce) in both groups, on average, suggesting there were no placebo effects resulting from either hidden or open placebo administration. This occurred despite significantly increased expectations of benefit in the “lorazepam” group following conditioning. If there were placebo effects present, then the size of these effects was small (d = 0.03 to 0.37) (Cohen, 1988). For comparison, 7 days of treatment with lorazepam reduces fearfulness during 7.5% CO_2_ inhalation with an effect size of d = 0.81 (Bailey et al., 2007), 21 days of paroxetine treatment reduces nervousness with d* *=* *0.92 (Bailey et al., 2007), and mindfulness techniques reduce state anxiety with partial η^2 ^=^ ^0.26 (Ainsworth et al., 2015). The placebo effects seen in the current study are unlikely to be meaningful or relevant compared with placebo effects seen in other experimental paradigms (Vase et al., 2002; Petrovic et al., 2005; Forsberg et al., 2017) and with standard anxiolytic treatments. These findings suggest that the CO_2_ inhalational model is relatively robust to expectations and beliefs. However, an exploratory analysis showed a trend-level negative relationship between expectations of benefit following conditioning and change in reactivity to CO_2_ challenge in the “lorazepam” group in subjective outcome measures only. Some participants, including some with high expectations, experienced an unexpected worsening of anxiety following conditioning (possibly consistent with a “nocebo response”: a worsening of health or development of new symptoms following administration of an inactive substance (Evers et al., 2018)).

There is only 1 other paradigm that has been developed for the explicit purpose of inducing placebo anxiolysis in healthy volunteers. Using an unpredictable threat of shock as the anxiogenic stimulus, a placebo accompanied by verbal suggestions that it was an anxiolytic drug (either “intranasal lorazepam” or “laughing gas”) reduced subjective fear and skin conductance responses in 3 related studies, regardless of threat (Meyer et al., 2015, 2019). Because the effect was not specific to threat trials, it is possible that the results represent another phenomenon, such as reduced attention, rather than reduced anxiety. Relatedly, placebo effects on “unpleasantness” of distressing images have been investigated in 2 studies (Petrovic et al., 2005; Guevarra et al., 2020), which might have overlaps with anxiety or feelings of threat. In the first of these studies, placebo effects were seen following a conditioning procedure involving active medications (midazolam and flumazenil) on day 1 and then a testing session on day 2 with saline only (Petrovic et al., 2005). In the second, an open-label placebo nasal spray reduced subjective distress in response to negative images but not neutral images in comparison with a control group (Guevarra et al., 2020).

The above studies share design features that differ from our design. First, experimenters were not blind and either involved in the deception of the participant or gave the placebo open-label. Instead, we chose to blind the experimenter to reduce the chance of response bias influencing the results (Hróbjartsson et al., 2011). However, it should be noted that in the open-label placebo study described above, effects were seen on a neural biomarker of distress (the late positive potential) even when participants were not asked to give feedback about their subjective feelings, suggesting perhaps that response bias is not the source of placebo effects (Guevarra et al., 2020). The other feature many of these studies share is a within-participants design, in which the placebo condition was compared with a control condition in the same individual (Petrovic et al., 2005; Meyer et al., 2015, 2019). A within-participants design can reduce the effect of inter-individual variability or confounders and increase signal to noise ratio (Sedgwick, 2014). We instead used a between-participants design, which might have increased noise and obscured the signal of the placebo effect. However, both groups were well balanced for potential confounders. Furthermore, between-group designs have been employed in many experimental placebo studies that demonstrated significant placebo effects (Watson et al., 2009; Huneke et al., 2013; Bartels et al., 2014; Glombiewski et al., 2019; Guevarra et al., 2020; Göhler et al., 2021). Finally, outcome measures in the current study were taken at a single timepoint post-conditioning after a relatively long duration aversive stimulus (20 minutes of 7.5% CO_2_ inhalation). Both pain and anxiety are subject to the peak-end rule, which states that participants are inclined to recall events mostly by how they were perceived at the experience’s peak and at its conclusion (Kahneman et al., 1993; Müller et al., 2019). The single post hoc measure used here might lack the sensitivity to detect placebo effects that occur earlier during the inhalation period. Additionally, repeated placebo administration could theoretically enhance its effects by reinforcing expectations and conditioning (Ashar et al., 2017). Nonetheless, at least 1 study has shown that placebo effects can be induced in tonic pain (through the cold pressor test) (Camerone et al., 2021). Overall, it appears these design choices alone do not explain the lack of consistent placebo effects in this study.

Another possibility to consider is that 7.5% CO_2_ inhalation might not be the most appropriate anxiogenic stimulus for a placebo conditioning paradigm. For example, inhalation of higher concentrations of CO_2_ (up to 35%) has been used to induce anxiety symptoms in healthy volunteers (Colasanti et al., 2008). Perhaps inhalation of a higher concentration might have increased the intensity of the anxiety experienced (Woods et al., 1988), allowing greater differentiation between the groups as a result of conditioning. Nevertheless, it should be noted that the *duration* of CO_2_ inhalation is also important, and an intense anxiety experience is known to be reliably induced with 20 to 30 minutes of 7.5% CO_2_ inhalation, as we have used here (Bailey et al., 2011; Garner et al., 2011; Ainsworth et al., 2015; Huneke et al., 2020b). Another consideration is that CO_2_ inhalation might affect anxiety regardless of placebo mechanisms such as expectations. The mechanisms through which inhalation of CO_2_ causes increased anxiety are not fully understood. An emerging hypothesis is that CO_2_-induced anxiety and fear-related behavior results from reductions in serum pH stimulating chemosensors (specifically acid-sensing ion channels) in brainstem serotonergic neurones, the amygdala, and the bed nucleus of the stria terminalis (Esquivel et al., 2010; Leibold et al., 2013; Taugher et al., 2014). The behavior, therefore, could be viewed as a mechanism to achieve homeostatic recalibration of pH (Leibold et al., 2015). Importantly, such behavior relies on midbrain and brainstem circuitry, rather than frontal circuitry, and results from a recent study suggest that the cognitive effects of CO_2_ inhalation extend to reductions in frontal executive functions. Healthy participants completed an intra-extradimensional set shift task and a spatial working memory task while undergoing 7.5% CO_2_ challenge. Compared with air inhalation, participants made more errors in these tasks in a pattern consistent with reduced prefrontal cortical function (Owen et al., 1991; Savulich et al., 2019). It is known that successful induction of placebo effects requires intact frontal cortical activity. For example, in patients with Alzheimer disease, reduced Frontal Assessment Battery scores and reduced functional connectivity of the prefrontal lobes with the rest of the brain are associated with reductions in the placebo component of analgesia (Benedetti et al., 2006). Additionally, when repetitive transcranial magnetic stimulation is used to transiently disrupt bilateral dorsolateral prefrontal cortex function, placebo analgesia is completely blocked in healthy volunteers (Krummenacher et al., 2010). It is possible that prefrontal cortical disruption because of 7.5% CO_2_ inhalation attenuated the placebo effect in the current study. Further studies are needed to understand how CO_2_ inhalation affects prefrontal cortex.

**Figure 4.**
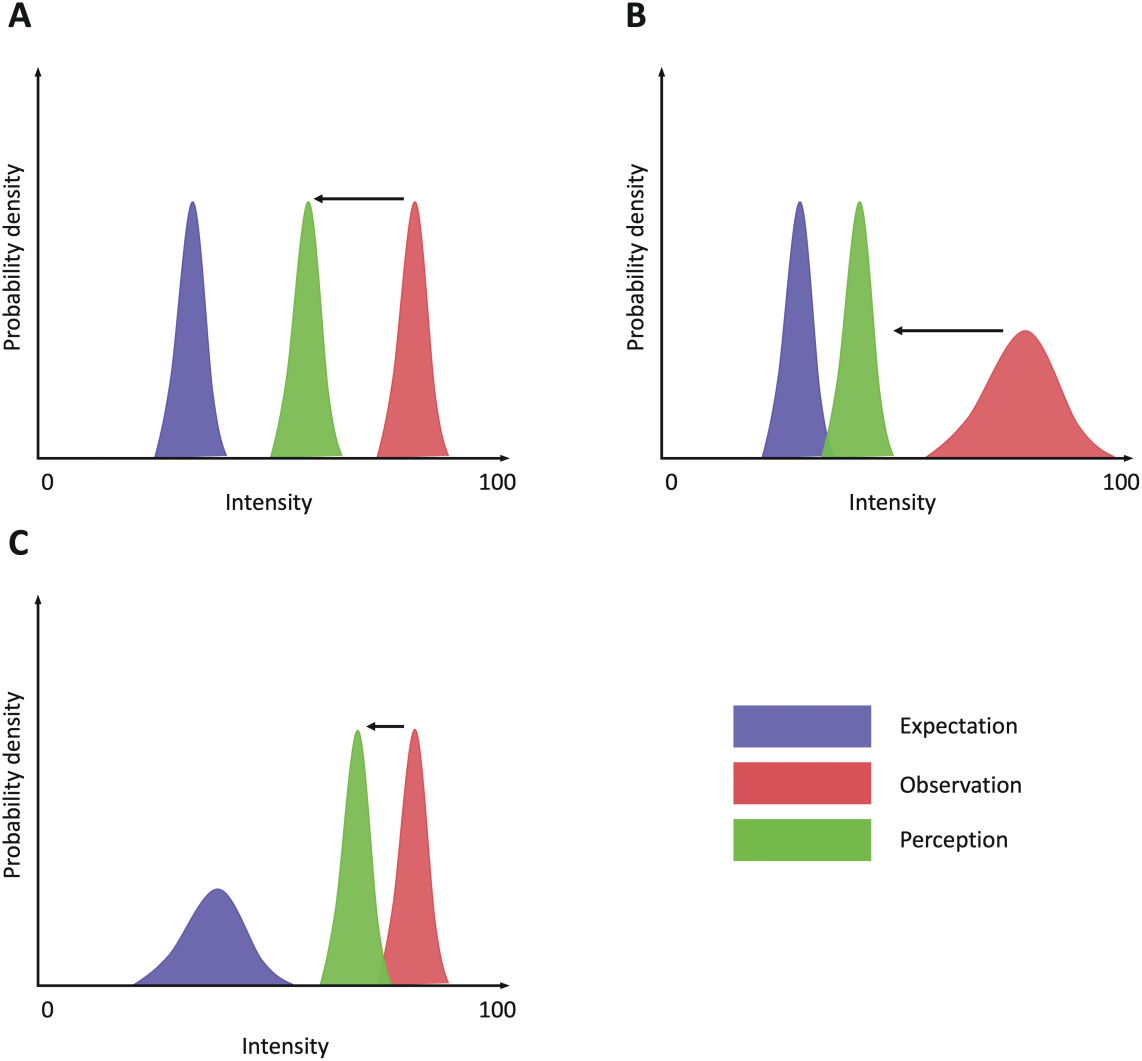
Cartoon demonstrating key concepts underpinning the Bayesian predictive coding framework of placebo effects. (A) The “prior” distribution (expectations) is compared with incoming sensory information (observation) and a decision is made that reconciles the 2 (perception). (B) Where incoming sensory information is noisy, then greater weight is given to the prior. (C) However, if there is uncertainty regarding expectations for future stimuli, then greater weight is given to incoming sensory information.

We additionally found a nonsignificant trend association between increased expectations post-conditioning and reduced sensitivity to CO_2_ challenge in subjective anxiety and mood measures only (see supplementary Material). However, some participants exhibited high expectations of benefit but did not experience a reduction in CO_2_ reactivity, instead showing no change or an increase. These findings can speculatively be explained through a Bayesian predictive coding framework, which has been offered as a potential model for understanding placebo effects (Büchel et al., 2014) (Figure 4). In this framework, it is thought that previous experience and conditioning are combined to form “a prior” (expectations). If a subsequent stimulus (“the posterior”) does not match the prior, this creates a prediction error. A Bayesian statistical inference follows, where the outcome is inferred to be somewhere between the prior prediction and the stimulus intensity. The difference between this posterior and the actual stimulus intensity represents the placebo effect (Büchel et al., 2014; Anchisi and Zanon, 2015). Crucially, in this framework, the prior expectations and ascending sensory signals are represented as probability density functions, meaning some level of certainty is encoded (Büchel et al., 2014). This is important, as it suggests that the “weighting” given to prior expectations or to ascending sensory evidence is determined by the relative certainty of each (Büchel et al., 2014; Grahl et al., 2018). For example, if there is uncertainty about the prior, then the posterior will be more closely aligned with the more certain incoming sensory information. However, if the incoming sensory information is highly variable, then the posterior might be more closely aligned with expectations. Indeed, this framework has been demonstrated to accurately model the effects of placebo conditioning on behavior (Anchisi and Zanon, 2015; Strube et al., 2023) and on neural signals in the peri-aqueductal grey matter (Grahl et al., 2018).

The concepts described by the Bayesian predictive coding framework might explain the current results in 2 ways. First, this framework suggests that placebo effects will be more pronounced when expectations are highly certain and when incoming sensory information is highly variable (Büchel et al., 2014; Grahl et al., 2018). In the current study, mean expectancy post-conditioning in the “lorazepam” group was 5.21 with the highest expectancy rating being 8.10 (maximum possible 10). This suggests that the participants were only moderately certain about their beliefs. The variability of sensory information during CO_2_ challenge is unknown. However, given that even 2 minutes of inhalation of 7.5% CO_2_ can induce significant anxiety (Pappens et al., 2012), and that test-retest reliability is high (Poma et al., 2005), it is likely the variability of this stimulus is low. In sum, the paradigm as designed could have led to low certainty regarding the anxiolytic effect of the placebo and high certainty regarding anxiogenic effects of CO_2_ challenge, a situation theorized to minimize placebo effects. Second, if incoming sensory information is “too different” from the prior model, then it is possible this model will be abandoned (Büchel et al., 2014). The conditioning phase of this study involved a low intensity stimulus (air inhalation). In the subsequent testing phase, participants again experienced a stimulus of high intensity (7.5% CO_2_). This might have led participants to question whether the medication was “working correctly.” Against this argument is the post-experiment measure of beliefs, which suggests the “lorazepam” group continued to hold positive expectations. However, as we did not ask participants about their experiences or whether they suspected any deception during or after the study, it is unclear whether such shifts in explanatory models could have attenuated the placebo effect.

### Limitations

This study had some limitations, most of which follow from the discussion above. First, the sample size of 32 was relatively small. Although this sample size should have been large enough to detect effect sizes seen in the experimental placebo literature, the numbers precluded any exploratory analyses of demographic or personality trait predictors of CO_2_ challenge outcome or of placebo effects. The study is also underpowered to detect smaller placebo effects, although it is unclear how relevant these effects would be. In addition, anxiety and mood were measured through a single questionnaire at the end of each inhalation. A continuous measure would have allowed quantification of the variation in subjective anxiety during a CO_2_ challenge and might have revealed interesting expectancy effects. Further, we did not collect subjective reports of how the participants experienced the placebo conditioning procedure. Reports regarding whether they suspected deception, and when, and how participants experienced the change from inhalation of CO_2_ to air and back to CO_2_ would have given valuable insights when appraising changes in CO_2_ reactivity, expectancy ratings, and the post-experiment therapeutic credibility questionnaire. Finally, it should be acknowledged that using verbal instructions to induce the placebo effect means that some aspects of this phenomenon might not generalize to clinical trial settings, where such manipulations do not take place. Research is also needed to determine whether mechanisms of placebo effects important in experimental approaches are associated with the mechanisms important in clinical trials.

## Conclusion

In summary, a conditioning paradigm using the 7.5% CO_2_ inhalational model of generalized anxiety disorder in healthy volunteers resulted in small placebo effects, on average. Both the placebo and control groups exhibited similar subjective and autonomic responses to CO_2_ challenge pre- and post-conditioning. The effect size of placebo was smaller than the effect size of active pharmacological and psychological treatments. These results support the use of the 7.5% CO_2_ model for evaluating potential anxiolytic effects of novel compounds in proof-of-principle studies. However, we additionally found a trend association between expectation and change in reactivity to CO_2_ challenge post-conditioning. Therefore, measurement of expectations and including these as a covariate could be considered for future studies using the 7.5% CO_2_ model. In addition, there was an unexpected group of participants that appeared to exhibit nocebo responses, despite positive expectations. It is possible that the design of the conditioning paradigm, the choice of anxiogenic stimulus, or behavior in line with a Bayesian predictive coding framework attenuated the effect of expectations on subsequent placebo response. Future studies should explore these possibilities.

## Supplementary Material

pyae019_suppl_Supplementary_Materials

## Data Availability

Data will be made available on the open science framework (https://osf.io/erdhy/).

## Interest Statement

D.S.B. is President of the British Association for Psychopharmacology and Editor of Human Psychopharmacology journal (for which he receives an editor’s honorarium). All other authors declare no conflicts of interest.
