# Supplementary material for: Placebo Effects Are Small on Average in the 7.5% CO2 Inhalational Model of Generalized Anxiety
Source: Int J Neuropsychopharmacol. 2024 Apr 5;27(4):pyae019. doi: 10.1093/ijnp/pyae019 (PMC11059817; doi:10.1093/ijnp/pyae019)

Placebo Effects Are Small on Average in the 7.5% CO_2_ Inhalational Model of Generalised Anxiety: Supplementary Material

Nathan TM Huneke, Cosmina Cross, Harry A Fagan, Laura Molteni, Naomi Phillips, Matthew Garner, David S Baldwin

**1. Supplementary Methods**

We carried out exploratory analyses including a *post-hoc* power analysis and an analysis examining whether expectations influenced outcome. First, given the effect sizes we saw in this study, we assessed the sample sizes needed to achieve 80% power to detect placebo anxiolysis using this experimental paradigm. Second, to explore the effect of expectations, we calculated the ‘CO_2_ reactivity’ for each participant in each inhalation by subtracting pre-inhalation values from post-inhalation values. Larger scores indicated a greater reactivity to CO_2_ challenge. We then calculated how this changed from pre-conditioning to post-conditioning by subtracting pre-conditioning reactivity from post-conditioning reactivity. Thus negative values represent a reduction in reactivity (potentially placebo anxiolysis) while positive values represent an increase in reactivity. We then correlated the change in CO_2_ reactivity for each outcome measure with expectations post-conditioning for each group separately.

**2. Supplementary Results**

**Table S1** Summary of mixed-model ANOVAs with repeated measures assessing CO2 outcomes. Significant results in bold.

| **Outcome Measure** | **Effect** | **Test statistic** | **p value** | **η_p_^2^** |
| --- | --- | --- | --- | --- |
| Subjective Anxiety and Mood |  |  |  |  |
| Modified GAD-7 | **Time**  Group  Time*Group | **F_(2.40,72.15)_ = 28.90**  F_(1,30)_ = 0.10  F_(2.40,72.15)_ = 0.40 | **< 0.001**  0.758  0.497 | **0.49**  0.00  0.02 |
| PANAS Positive Affect | **Time**  Group  Time*Group | **F_(2.26,67.83)_ = 38.72**  F_(1,30)_ = 0.10  F_(2.26,67.83)_ = 1.07 | **< 0.001**  0.752  0.353 | **0.56**  0.00  0.03 |
| PANAS Negative Affect | **Time**  Group  Time*Group | **F_(1.77,51.39)_ = 15.25**  F_(1,29)_ = 0.24  F_(1.77,51.39)_ = 0.46 | **< 0.001**  0.631  0.608 | **0.34**  0.01  0.02 |
| PSI | **Time**  Group  Time*Group | **F_(1.80,54)_ = 25.92**  F_(1,30)_ = 0.08  F_(1.80,54)_ = 0.03 | **< 0.001**  0.785  0.959 | **0.46**  0.00  0.00 |
| Autonomic Measures |  |  |  |  |
| Systolic BP (mmHg) | **Time**  **Group**  Time*Group | **F_(3,90)_ = 5.87**  **F_(1,30)_ = 5.24**  F_(3,90)_ = 0.60 | **0.001**  **0.029**  0.620 | **0.16**  **0.15**  0.02 |
| Diastolic BP (mmHg) | Time  Group  Time*Group | F_(3,90)_ = 0.17  F_(1,30)_ = 0.86  F_(3,90)_ = 1.03 | 0.914  0.362  0.381 | 0.01  0.03  0.03 |
| Pulse rate (bpm) | **Time**  Group  Time*Group | **F_(1.70,50.87)_ = 13.46**  F_(1,30)_ = 1.22  F_(1.70,50.87)_ = 0.94 | **< 0.001**  0.278  0.383 | **0.31**  0.04  0.03 |
| Abbreviations: GAD-7, Generalised Anxiety Disorder-7 questionnaire; PANAS, Positive and Negative Affect Schedule; PSI, Panic Symptoms Inventory; BP, Blood pressure; mmHg, millimetres of mercury; bpm, beats per minute. | | | | |

**Table S2** Summary of effect sizes for post-hoc between-group comparisons of subjective and autonomic outcome measures following the second CO2 inhalation and sample size required for 80% power to detect the effect. Where mean difference is positive, this indicates a larger value for the ‘lorazepam’ group.

| **Variable** | **Mean Difference** | **CI_95%_** | **Test result** | **Cohen’s *d*** | ***N* per group required** |
| --- | --- | --- | --- | --- | --- |
| Modified GAD-7 | 2.83 | -14.09, 19.75 | t_(30)_ = 0.34, p = 0.735 | 0.12 | 1092 |
| PANAS Positive Affect | -0.40 | -7.15, 6.34 | t_(30)_ = 0.12, p = 0.903 | 0.04 | 9813 |
| PANAS Negative Affect | 0.51 | -2.89, 3.90 | t_(30)_ = 0.30, p = 0.763 | 0.11 | 1299 |
| PSI | -1.04 | -13.11, 11.04 | t_(30)_ = 0.18, p = 0.862 | 0.06 | 6281 |
| Systolic BP (mmHg)* | 1.92 | -6.30, 10.14 | t_(29)_ = 0.48, p = 0.636 | 0.19 | 436 |
| Diastolic BP (mmHg) | -0.18 | -6.36, 6.00 | t_(30)_ = 0.06, p = 0.953 | 0.02 | 39246 |
| Pulse rate (bpm) | -7.34 | -21.87, 7.19 | t_(30)_ = 1.03, p = 0.311 | 0.37 | 116 |
| *Pre-inhalation 1 systolic BP included as a covariate, due to significant difference between groups Abbreviations: CI_95%_, 95% confidence interval; GAD-7, Generalised Anxiety Disorder-7 questionnaire; PANAS, Positive and Negative Affect Schedule; PSI, Panic Symptoms Inventory; BP, Blood pressure; mmHg, millimetres of mercury; bpm, beats per minute. | | | | | |


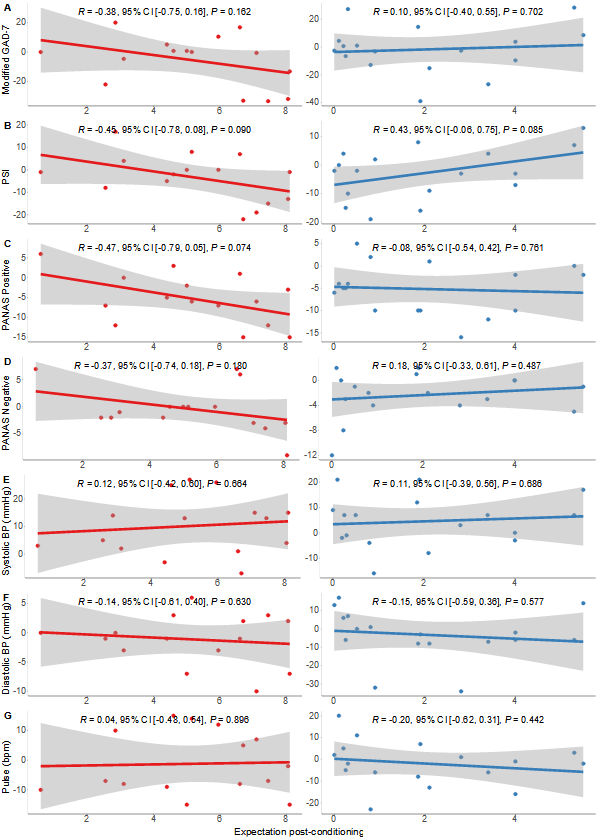


**Figure S1:** Correlations between change in CO_2_ reactivity from pre- to post-conditioning in each outcome measure and expectation post-conditioning computed for each group separately (left: ‘lorazepam’; right: ‘saline’). Negative values on the y axis represent reduced CO2 reactivity post-conditioning. There was a trend negative relationship between change in CO_2_ reactivity in subjective measures of anxiety and expectation post-conditioning in the ‘lorazepam’ group only. Abbreviations: GAD-7, Generalised Anxiety Disorder-7 questionnaire; PANAS, Positive and Negative Affect Schedule; PSI, Panic Symptoms Inventory; BP, Blood pressure; mmHg, millimetres of mercury; bpm, beats per minute.

**
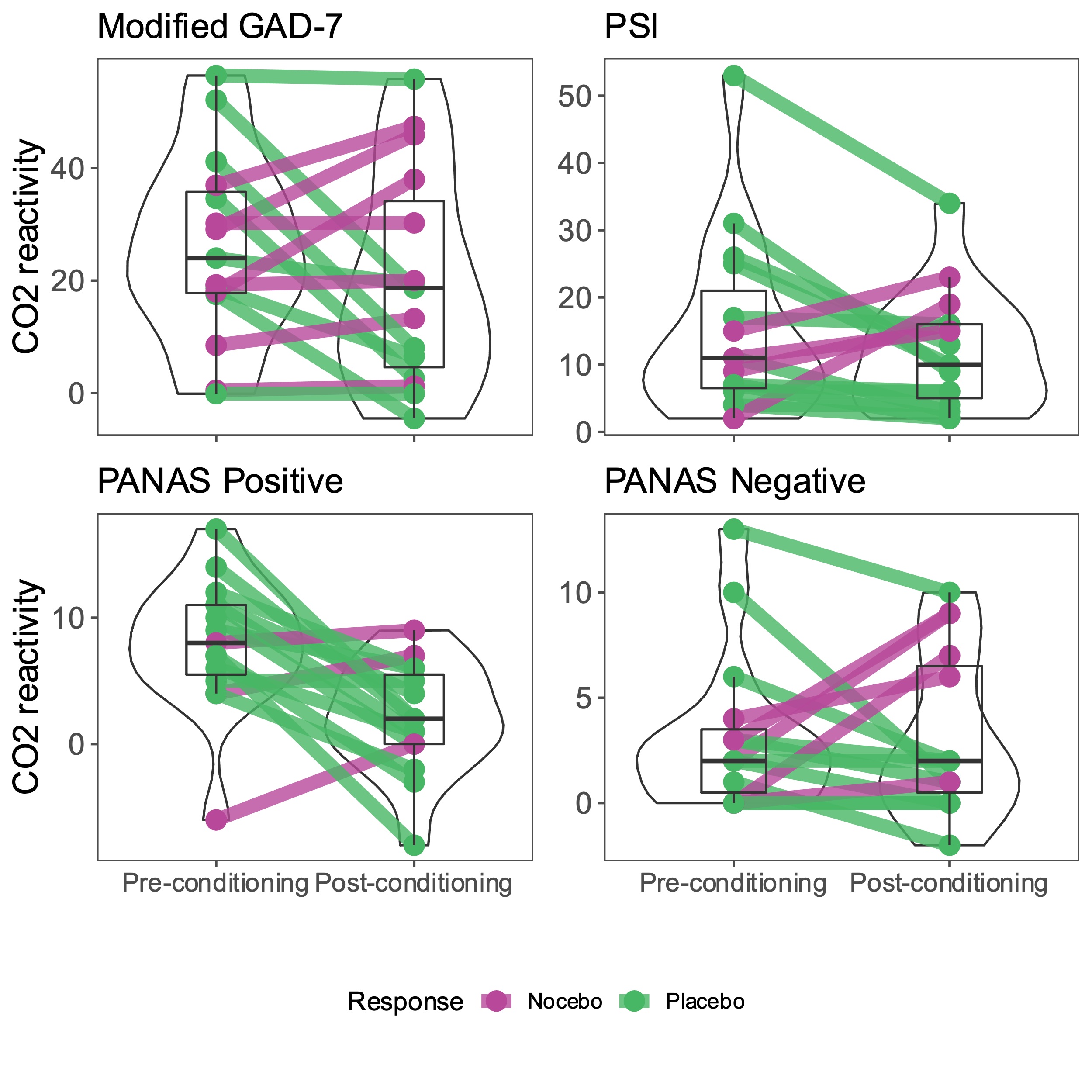
Figure S2**: Change in CO_2_ reactivity from pre- to post-conditioning in subjective CO_2_ outcome measures for the ‘lorazepam’ group. Negative values on the y axis represent reduced CO_2_ reactivity. In blue are participants who showed increases in CO_2_ reactivity post-conditioning, consistent with a nocebo response. In red are participants who showed reductions, consistent with a placebo response. Abbreviations: GAD-7, Generalised Anxiety Disorder-7 questionnaire; PANAS, Positive and Negative Affect Schedule; PSI, Panic Symptoms Inventory.

***Figure S3:*** *Graphs showing change in expectations, and in anxiety, mood and autonomic CO2 outcome measures over the course of the experiment. Points represent estimated marginal means and error bars represent 95% confidence intervals. The 'lorazepam' group is shown in red and the 'saline' group is shown in blue. Abbreviations: GAD-7, Generalised Anxiety Disorder-7 questionnaire; VAS, visual analogue scale; PANAS, Positive and Negative Affect Schedule; PSI, Panic Symptoms Inventory; BP, Blood pressure; mmHg, millimetres of mercury; bpm, beats per minute*


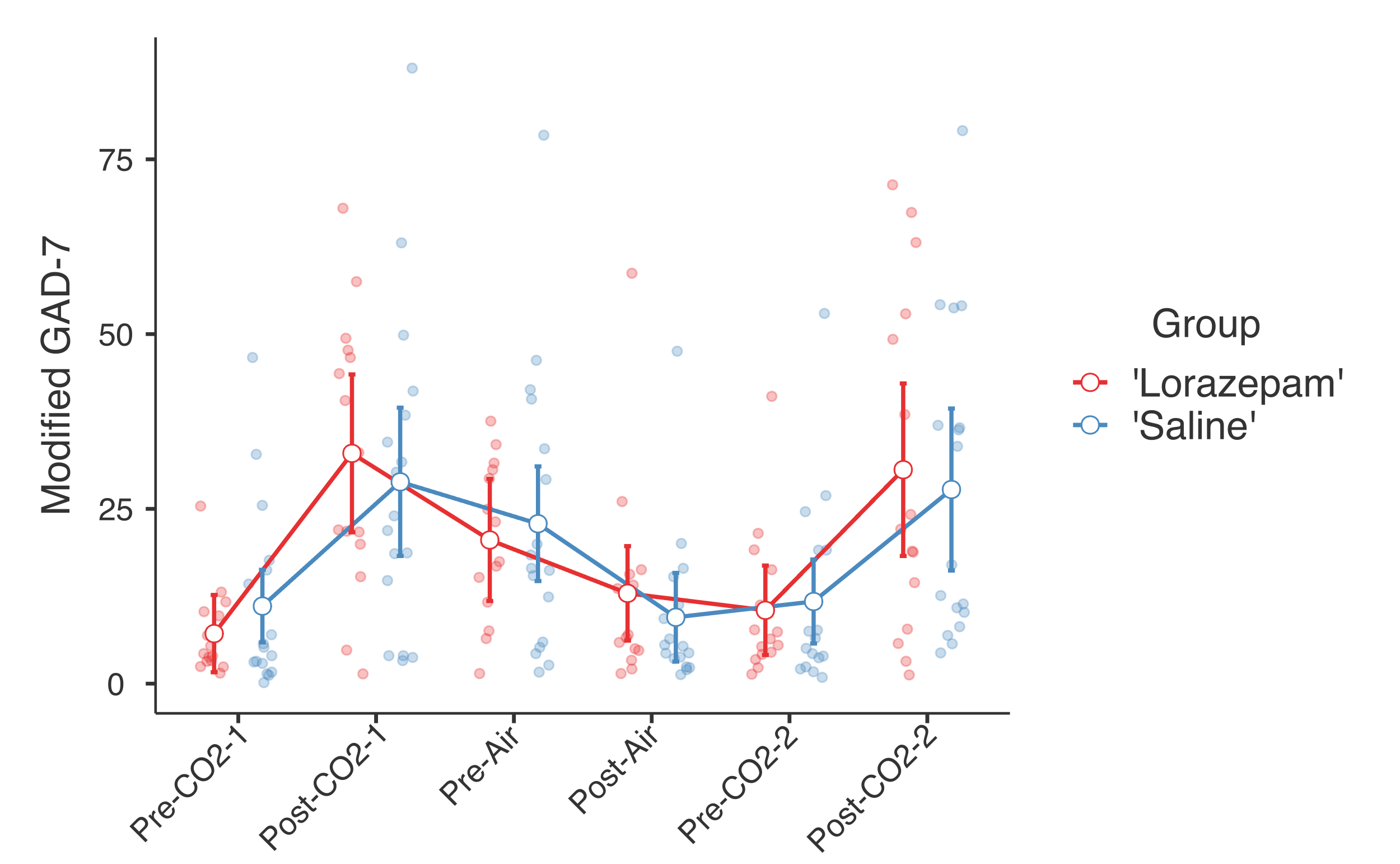


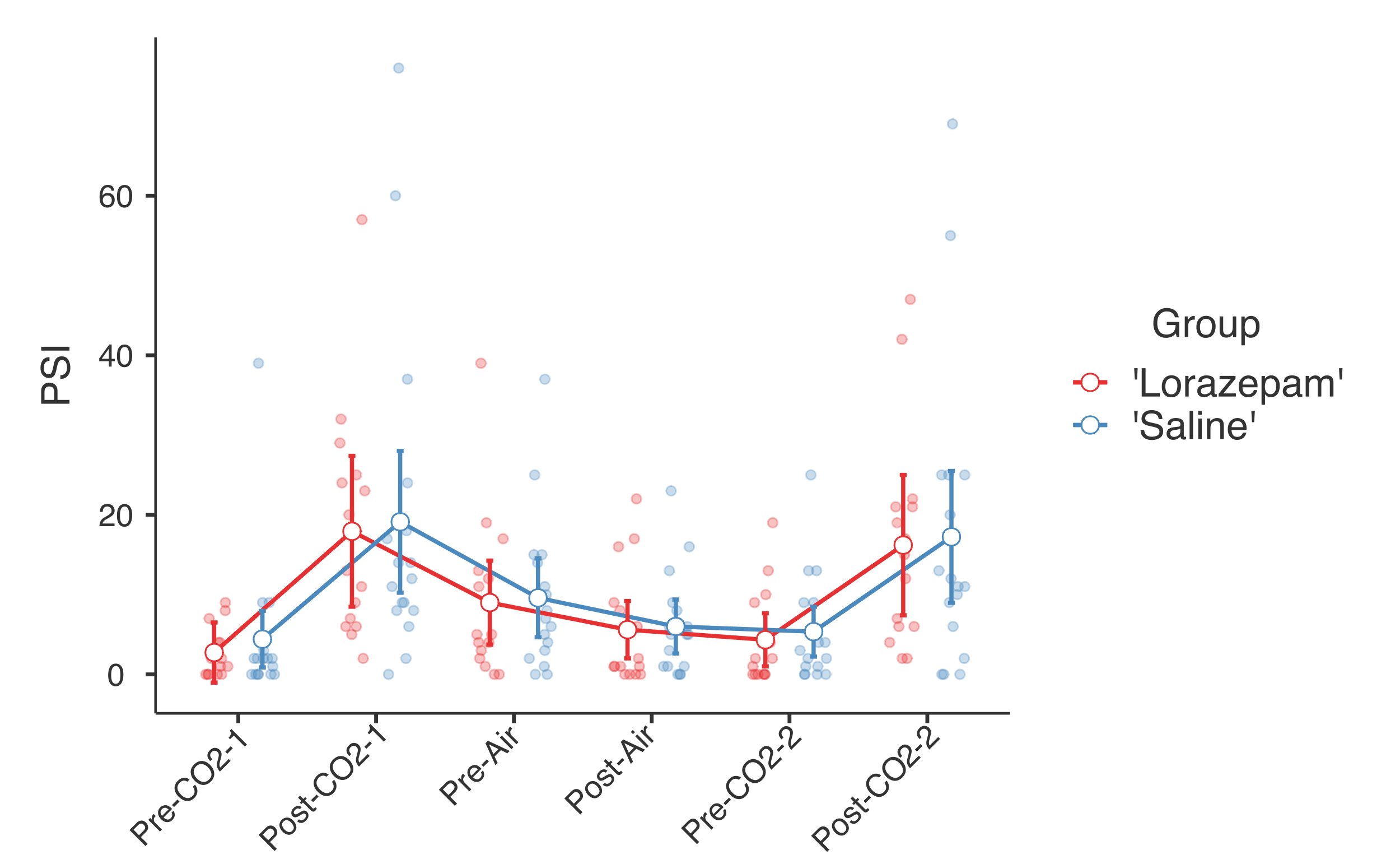


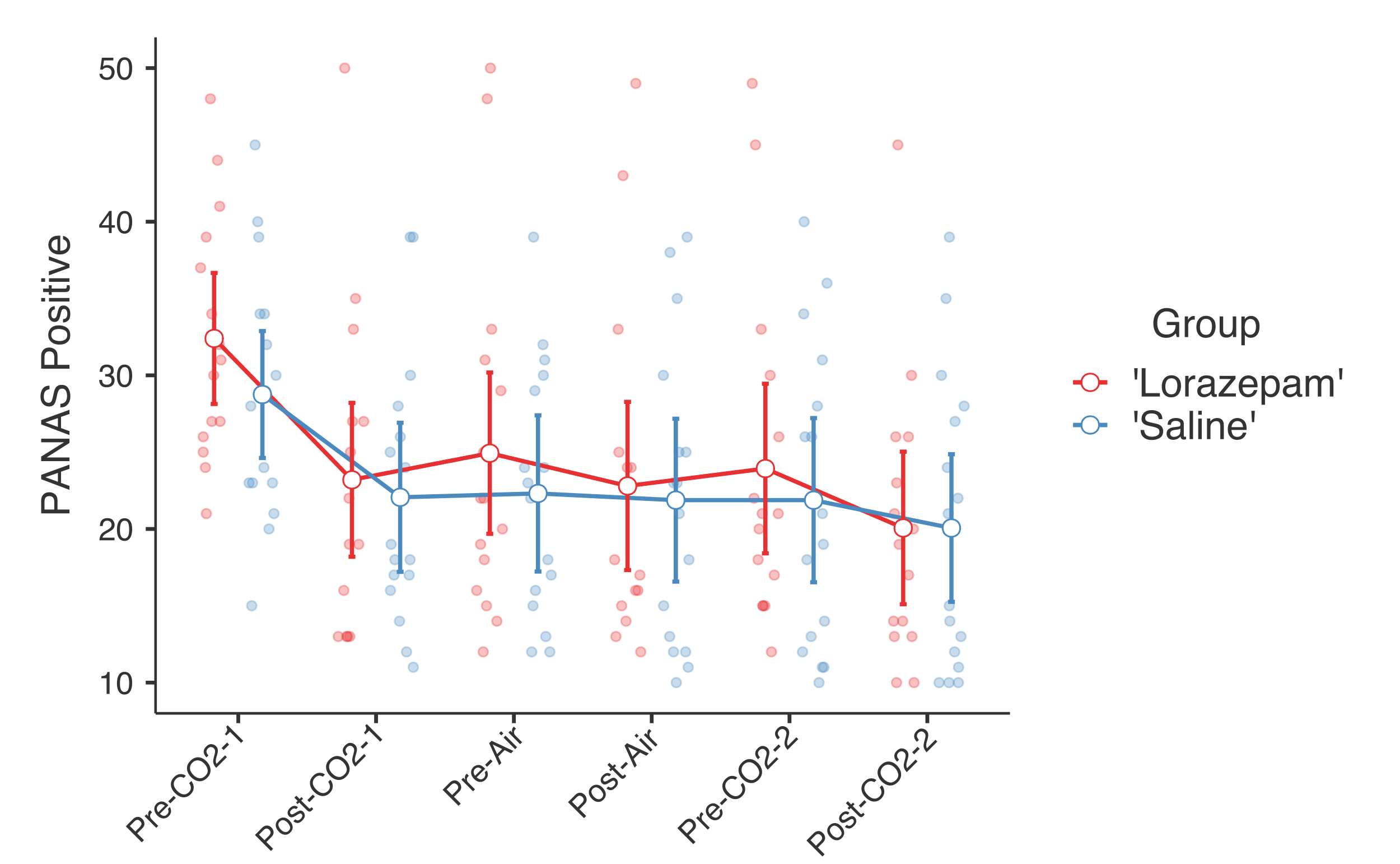


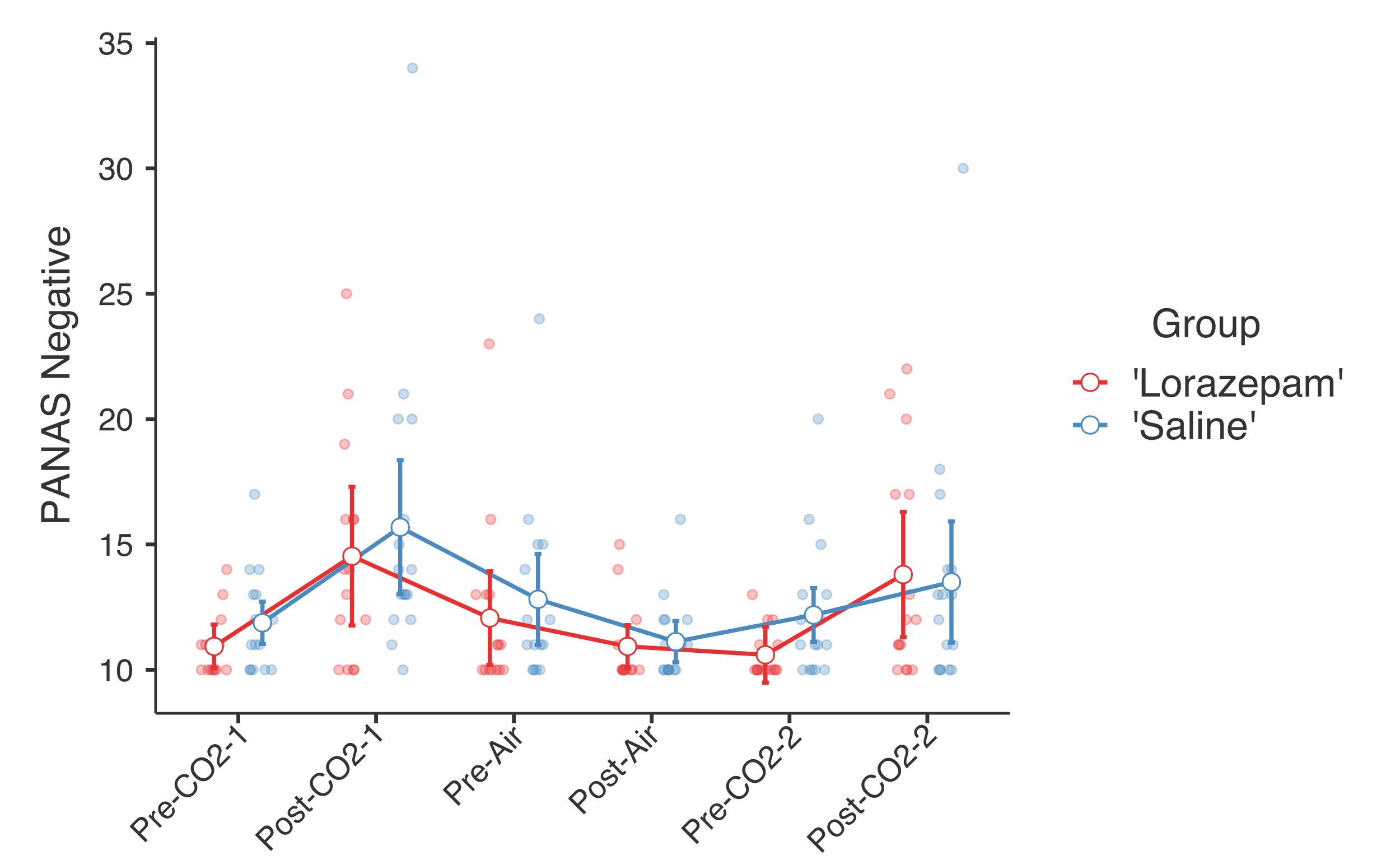


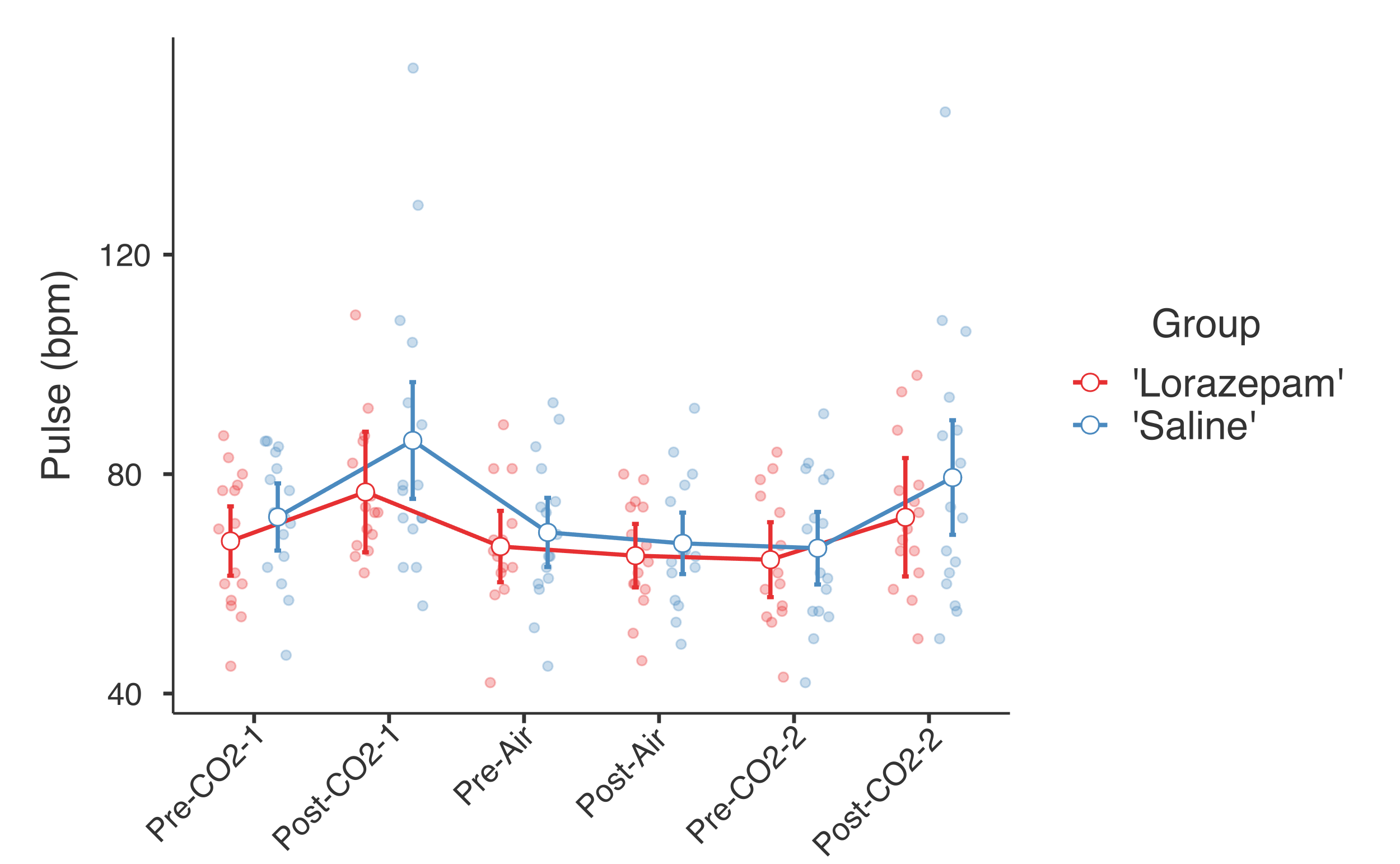


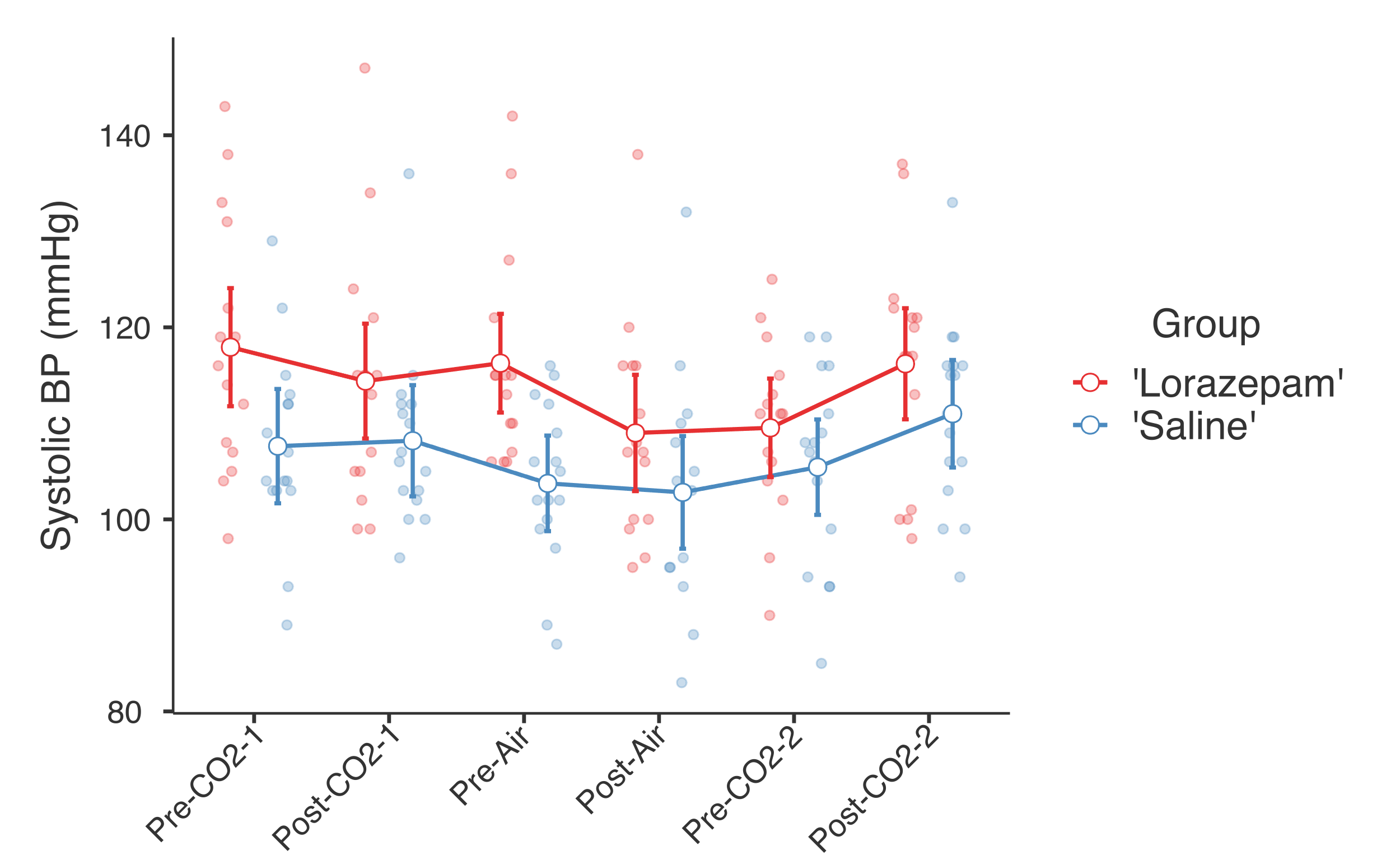


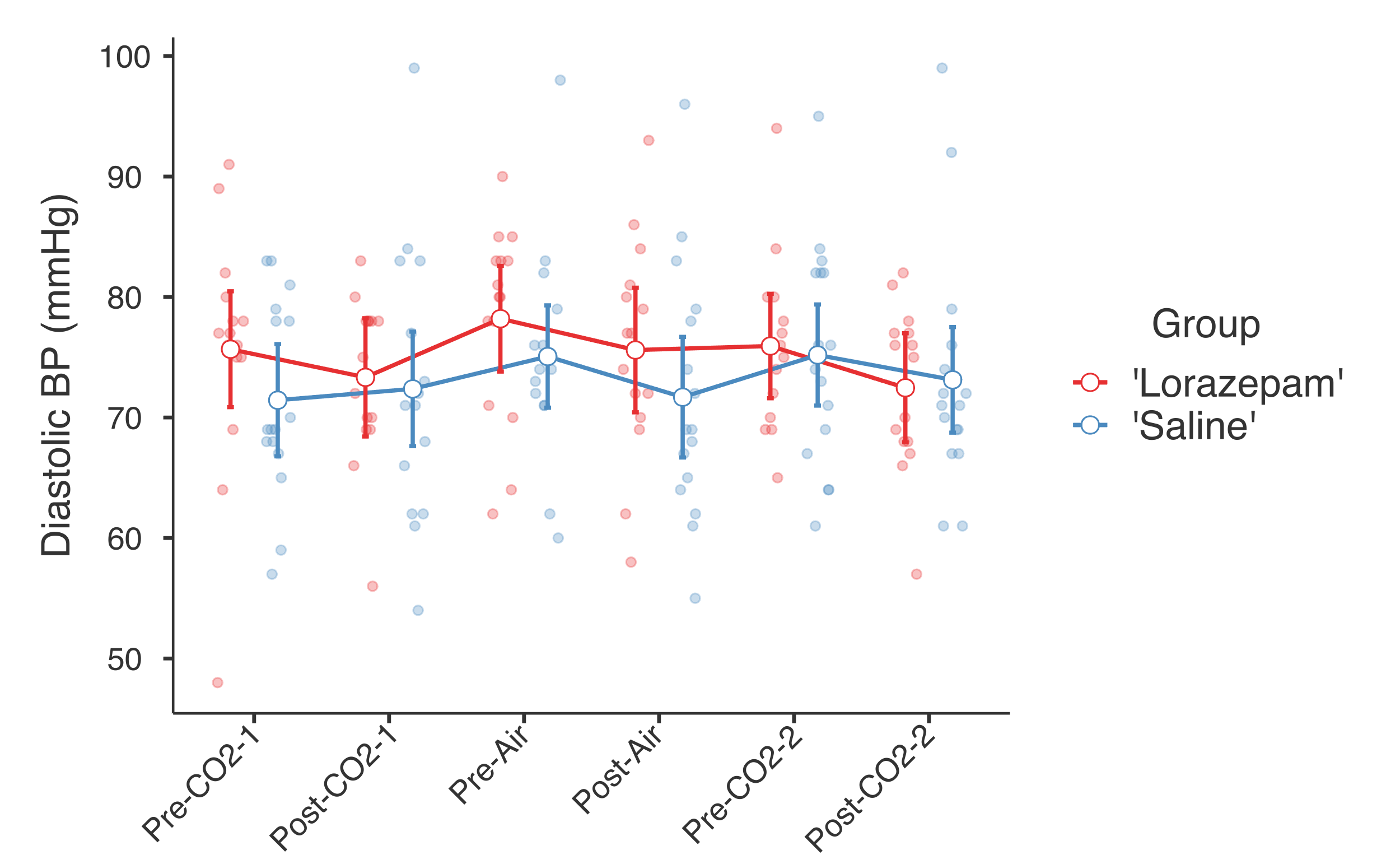

Supplement: pyae019_suppl_Supplementary_Materials [file pyae019_suppl_supplementary_materials.docx]
